# Utility and First Clinical Application of Screening Embryos for Polygenic Disease Risk Reduction

**DOI:** 10.3389/fendo.2019.00845

**Published:** 2019-12-04

**Authors:** Nathan R. Treff, Jennifer Eccles, Lou Lello, Elan Bechor, Jeffrey Hsu, Kathryn Plunkett, Raymond Zimmerman, Bhavini Rana, Artem Samoilenko, Steven Hsu, Laurent C. A. M. Tellier

**Affiliations:** ^1^Genomic Prediction Inc., North Brunswick, NJ, United States; ^2^Genomic Prediction Clinical Laboratory, North Brunswick, NJ, United States; ^3^Department of Physics and Astronomy, Michigan State University, East Lansing, MI, United States

**Keywords:** polygenic risk score, preimplantation genetic testing, advanced maternal age, aneuploidy, type 1 diabetes

## Abstract

For over 2 decades preimplantation genetic testing (PGT) has been in clinical use to reduce the risk of miscarriage and genetic disease in patients with advanced maternal age and risk of transmitting disease. Recently developed methods of genome-wide genotyping and machine learning algorithms now offer the ability to genotype embryos for polygenic disease risk with accuracy equivalent to adults. In addition, contemporary studies on adults indicate the ability to predict polygenic disorders with risk equivalent to monogenic disorders. Existing biobanks provide opportunities to model the clinical utility of polygenic disease risk reduction among sibling adults. Here, we provide a mathematical model for the use of embryo screening to reduce the risk of type 1 diabetes. Results indicate a 45–72% reduced risk with blinded genetic selection of one sibling. The first clinical case of polygenic risk scoring in human preimplantation embryos from patients with a family history of complex disease is reported. In addition to these data, several common and accepted practices place PGT for polygenic disease risk in the applicable context of contemporary reproductive medicine. In addition, prediction of risk for PCOS, endometriosis, and aneuploidy are of particular interest and relevance to patients with infertility and represent an important focus of future research on polygenic risk scoring in embryos.

## Introduction

Contemporary preimplantation genetic testing (PGT) is a well-established method for reducing the risk of adverse outcomes in *in vitro* fertilization (IVF). PGT-A (aneuploidy screening) reduces the risk of miscarriage, implantation failure, and multiples without compromising success rates (1, 2). Given the maternal age-related increase in aneuploidy, PGT-A has also been shown to be particularly important in older women (3). PGT-SR (structural rearrangements) is also widely used by couples which carry a balanced translocation or other structural rearrangement, in order to reduce the risk of miscarriage and to prevent disease associated with an unbalanced karyotype (4). PGT-M (monogenic disease) has also helped prevent many serious conditions in children born to at risk parents (5). Finally, as Edwards and Schulman predicted in 1996, testing embryos for polygenic disorders is now possible (6). As with any new application in reproductive medicine, the recent introduction of PGT-P (for polygenic disease risk reduction) has been met with both criticism and enthusiasm.

A recent study suggested that screening embryos for polygenic traits has limited utility (7). The authors elected to define utility as *expected gain* in trait value. However, there may also be utility in eliminating negative (disease) outcomes, which is arguably a more ethical and practical application of polygenic risk scoring in human embryos. In other words, utilization of PGT-P to reduce disease risk, as opposed to improving upon a desired trait, may be more ethically justifiable, as well as more likely to be successful. Interestingly, an American Society for Reproductive Medicine (ASRM) Practice Committee opinion on PGT for adult onset conditions of lesser severity states that “testing is not available for multifactorial diseases” (8).

The first step in making such testing (PGT-P) possible, is demonstrating the ability to accurately obtain genome-wide genotypes from an embryo biopsy. This step has been completed, with >99% concordance between limited quantities of DNA when compared to large quantities of DNA (9). As a result of this advance, it is now possible to equate performance of polygenic risk scoring in adult populations to performance on embryos produced during IVF. Khera et al. has already shown that many polygenic diseases, including breast cancer, type 2 diabetes, coronary artery disease, and atrial fibrillation can be accurately predicted in adults, “with risk equivalent to monogenic mutations” (10). DNA databases with sibling cohorts are also now available, and allow for analysis of the risk reduction provided by genetic testing for selection against polygenic disease.

For example, 2,601 type 1 diabetes affected/unaffected, sibling-pair families are available from the type 1 diabetes consortium (T1DGC) (11). These data provide an opportunity to evaluate relative risk reduction by comparing randomly selected siblings to siblings selected based on having the lowest polygenic risk score. Herein we report performance of PGT-P in the context of diabetes risk reduction by genetic testing of multiple siblings. We also provide initial observations from the first clinical application of PGT-P in families with a history of complex disease.

## Materials and Methods

### Mathematical Model

The liability threshold model is widely used for binary disease traits in genetics. In this model, disease status is completely determined by a continuous liability score exceeding a threshold. To generate a realistic model for a group of siblings, a correction for sibling relatedness was performed. Recent papers have shown that using logistic regression on HLA risk haplotypes in addition to other genome-wide significant loci show strong out-of-sample predictive validity for type 1 diabetes (12, 13). These constructed genomic risk scores achieved a 0.85–0.92 area under the curve for type 1 diabetes out-of-sample validation on the UK biobank (14).

Here, 2 additional predictors were implemented to test the affected sibling pair cohort of the type 1 diabetes consortium (T1DGC). Oram et al. (13), which uses the Barker et al. (12) methodology to determine the high-risk HLA MHC (DR3/DR4-DQ8, DR3/DR3, etc.) haplotypes (DRB1-DQA1-DQB1 gene locus), and Sharp et al. (14), which uses SNPs with high r2 to tag for the specific haplotypes as well as modeling interaction effects between haplotypes.

SNPs were lifted over to hg19/GRcH37 using liftOver (15) and SNP flips were handled using conform-gt (https://faculty.washington.edu/browning/conform-gt.html). Samples were imputed using BEAGLE v5 to 1KG data (16). In addition, affected sibling pair samples were sent to University of Michigan's HRC imputation server (https://imputationserver.sph.umich.edu/index.html#!) and imputed. Missing genotypes from the 1KG imputation were filled in with the HRC imputation.

For relative risk reduction analysis, siblings were randomly sampled (100 times) and compared with taking the lowest polygenic risk score sibling. The T1DGC first collected affected sibling pair families from four geographic networks (Asia-Pacific, Europe, North America, United Kingdom), and ancestry analysis was performed upon this cohort, confirming a mixture of Caucasian and East Asian ancestry components. In addition, type 1 diabetes cases and controls were ascertained from existing and *de novo* collections. The T1DGC assembled 2,601 type 1 diabetes affected sibling pair families. Additional information can be found in Supplementary Methods.

### Clinical Case

**Figure 1:**
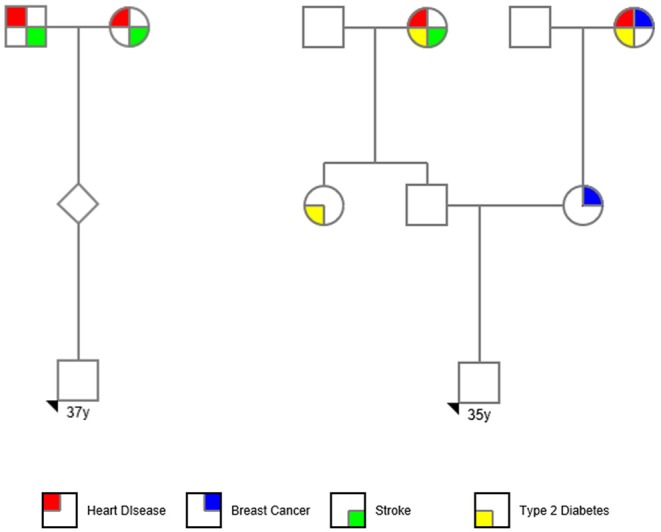
PGT-P case pedigree.

A same-sex couple was referred to Genomic Prediction Clinical Laboratory (CLIA#31D2152380) for PGT-P. The couple reported a family history of heart disease and stroke in one set of grandparents in one male partner (age 37), and a history of breast cancer (mother and paternal grandmother), and type 2 diabetes (maternal grandmother, paternal grandmother, and paternal aunt) in the other partner (age 35). The couple otherwise denied a personal or family history of other polygenic conditions available for testing at Genomic Prediction Clinical Laboratory. The couple denied a history of multiple pregnancy loss, parental chromosome rearrangements, previous pregnancies, or family history of aneuploidy. Standard pedigree nomenclature (17) was used in Figure 1. The couple chose to use an oocyte donor (age 23) and to fertilize half with one partner's sperm and half with the others. The couple consented to research.

### Genetic Counseling

The following text describes information provided to patients during genetic counseling and prior to obtaining informed consent for performing PGT-P.

PGT-P refers specifically to screening embryos for one or more polygenic disorders; diseases influenced by genetic variants in more than one gene. The purpose of this testing is to identify which embryos have an increased lifetime risk of developing specific disease conditions, such as type I diabetes and coronary artery disease. Embryo biopsy samples are evaluated for hundreds of thousands of single nucleotide polymorphisms (SNPs) to produce data that can be used to define a polygenic risk score for the specific disease(s) of interest. Each PGT-P predictor is trained on a large repository of hundreds of thousands of genomes with associated clinical phenotypes, as part of validating a polygenic risk score (PRS) model.

Validation on positive controls demonstrated a diagnostic accuracy of 94% for PGT-P on the polygenic trait of type 1 diabetes, and a variant concordance with the controls which exceeded 99% for PGT-P genotyping. The genotyping concordance refers to the ability of the test to correctly characterize genetic variants using the PGT-P genotyping platform. The clinical positive predictive value (prediction of a clinical diagnosis of a given disease) varies from disease trait to disease trait.

Saliva samples from both biological parents are required as part of this analysis to aid in data interpretation for improved accuracy.

A “high risk” result indicates that the polygenic risk score computed from the embryo's genotype suggests a high risk for the disease whose risk is being assessed. Empirical studies of individuals with equivalent polygenic risk scores indicate a lifetime disease risk where the average population-matched individual with this score is in the top 2% of risk.

A “normal risk” result indicates that the polygenic score computed from that embryo's genome does not indicate risk exceeding the top 2% of genomes in that individual's population. Each embryo is in a “risk percentile,” which describes the fraction of other genomes in their population which is lower than them in polygenic risk. In this way, a 98th percentile embryo is in the top 2% of highest risk. Generally, a lower risk (and a lower percentile) is preferable.

An “inconclusive” result indicates that the data could not be interpreted to determine polygenic risk scores. The chance for an embryo to be designated as inconclusive is <1%. A “no amp” result indicates that the no DNA was detected in the sample. The chance for an embryo to be designated as no amp is <5%. In these scenarios, a repeat biopsy is usually recommended to complete the analysis.

PGT-P results are based upon polygenic risk scores (PRS) for each given disease trait. PGT-P is a screening tool, designed to provide a risk estimate only. This is not a diagnostic test. PRS are not a guarantee of the presence or absence of disease. PGT-P is designed to provide a PRS only for the specific condition(s) requested by the ordering provider. Additional PRS for other polygenic diseases are not included in analysis. In demographics different from the Caucasian training set, sensitivity will be reduced.

Disease risk is adjusted to the sex of the embryo and the familial disease history, where available. For most polygenic conditions, disease risk will be additionally influenced by environmental, and other non-genetic factors. PGT-P does not address these factors as part of the analysis. Less frequently, conditions that are generally considered polygenic may be highly affected by rare, monogenic variants, which are inherited in certain families. If these variants are rare, the polygenic risk score may not take these into account, and the polygenic risk score accuracy may perform with severely reduced prediction of true disease risk. Based on these limitations, testing for the polygenic disorder(s) in individuals conceived following PGT-P testing is recommended according to standard clinical criteria.

PGT-P is not a replacement for PGT-M and is not capable of detecting monogenic causes of disease. For cases with known monogenic variants causing a Mendelian inheritance pattern of disease, these variants should be addressed using PGT-M.

Additional polygenic disorders, monogenic disorders, microdeletion and microduplication syndromes, segmental aneuploidies associated with parental chromosome rearrangements, and other genetic and non-genetic disorders, are outside the scope of PGT-P screening, and will not be detected by this test.

## Results

### Type 1 Diabetes Risk Reduction

**Figure 2:**
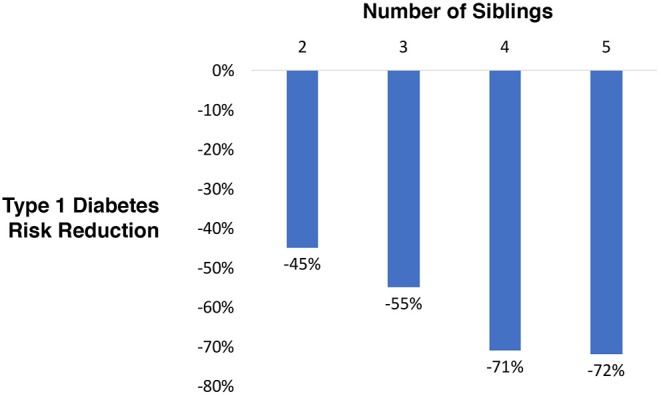
Relative type 1 diabetes risk reduction when selecting a sibling using blinded genetic testing for polygenic scoring compared to random selection.

A type 1 diabetes high risk cohort (families with history of the disease) was evaluated. There were families with 2, 3, 4, and 5 siblings, all fully grown adults, with and without mature (final) type 1 disease status. The probability of randomly selecting a sibling with type 1 diabetes was compared to the probability after ranking siblings by genetic testing for polygenic risk. A reduction of 45% for 2 siblings, 55% for 3, 71% for 4, and 72% for 5 was observed (Figure 2).

### PGT-P Case

**Figure 3:**
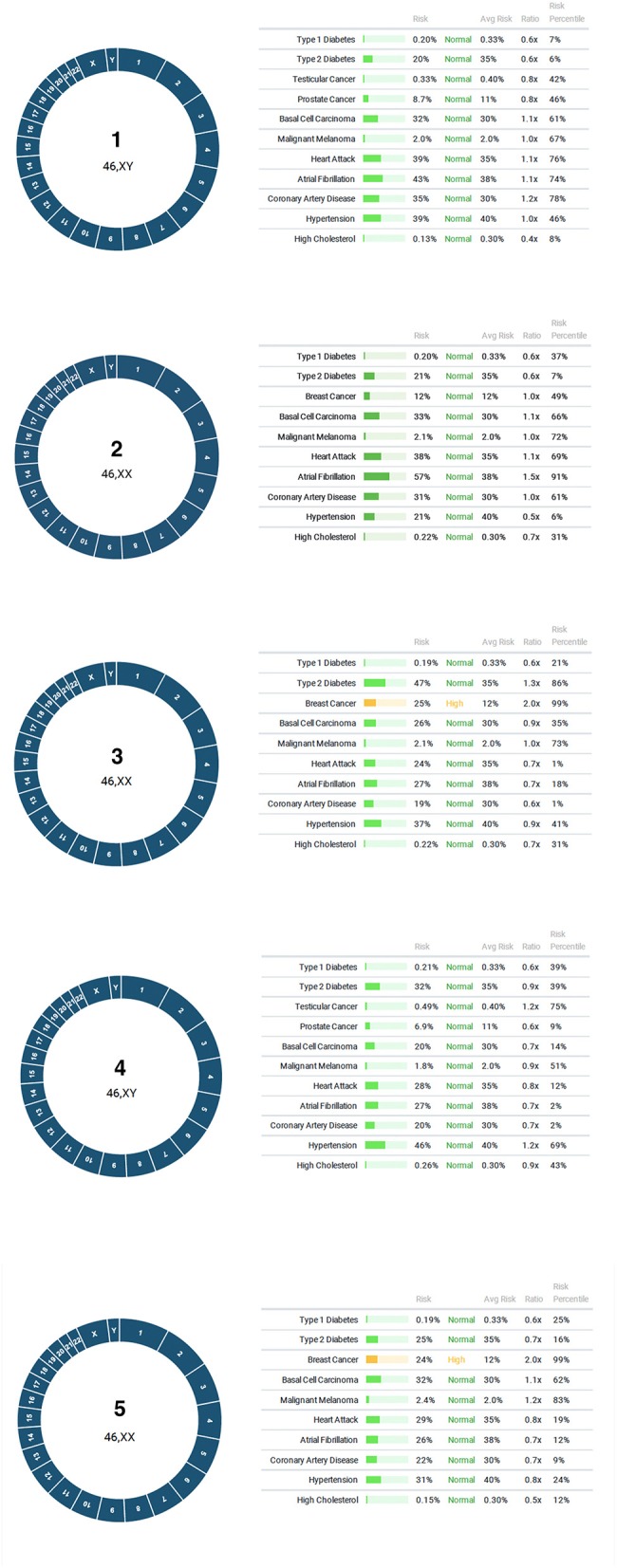
Results of PGT-P. All five embryos were euploid, with two displaying high risk for breast cancer.

In the first application of PGT for polygenic disease risk, 6 embryos were tested for aneuploidy and for lifetime risk of type 1 diabetes, type two diabetes, breast cancer, testicular cancer, prostate cancer, basal cell carcinoma, malignant melanoma, heart attack, atrial fibrillation, coronary artery disease, hypertension, high cholesterol. One embryo gave inconclusive results due to failed amplification. A repeat biopsy was recommended. All five remaining embryos were euploid, and two displayed a high risk for breast cancer (Figure 3). The couple elected to perform another cycle before proceeding with embryo transfer to a gestational carrier.

## Discussion

This study demonstrates the clinical utility of PGT for type 1 diabetes risk reduction, ranging from 45 to 72% in families with an affected individual. The model accounts for having 2–5 siblings available for genetic testing. Interestingly, patients utilizing PGT-M commonly produce ~6–7 embryos following IVF (18). In addition, patients utilizing PGT-M often do so based on family history (19). The model presented here is analogous to this situation, in that diabetes risk reduction was applied by genetic testing of siblings where the family was known to have a history of type 1 diabetes.

It is also reasonable to expect that patients seeking infertility treatment for other indications may produce 2 or more euploid embryos. In the first case involving PGT-P, the couple produced five euploid blastocysts and presented with a family history of several polygenic disorders, including breast cancer. Two embryos were found to have a high risk of breast cancer (top 99th percentile of risk, or higher) with >2X the average risk. A unique feature of the PGT methodology performed in this study is the ability to perform testing in all four major categories (PGT-A, -M, -SR, and -P) using the same platform. As a result of this capability, the additional information on polygenic risk (i.e., cancer), could be performed at no additional cost to the patient. This illustrates an important opportunity for patients to elect to obtain PGT-P results *after* they have determined the number of euploid blastocysts available for transfer.

Despite widespread application and demonstrated clinical utility, PGT has spurred numerous debates regarding the ethics of its use (6, 8, 20–22). Controversial applications include savior siblings, social sexing, adult onset disease testing, and testing for diseases of lesser severity and penetrance.

Data on PGT for social sexing (PGT-SS), also referred to as family balancing, was recorded by the European Society for Human Reproduction and Embryology PGT Consortium for over a decade before the procedure was deemed “ethically unacceptable” (23). PGT-SS involves elective IVF (i.e., treatment of patients without infertility), embryo biopsy, genetic testing to determine each embryos sex, and selection of the desired sex for embryo transfer. While the percentage of PGT-SS was routinely reported to represent only 2% of all PGT applications, many patients are able to choose the sex of the embryo for transfer as a result of incidental testing during conventional PGT.

Although some outlying countries and IVF programs still debate clinical utility, it is generally accepted that patients with advanced maternal age be offered the option of PGT-A to reduce the risk of miscarriage due to aneuploidy. An ethics committee of the American Society for Reproductive Medicine has recently published an opinion on disclosing an embryo's sex when incidentally revealed as part of the PGT process (22). Essentially, the committee argues that each clinic should create a formal policy, and that patients should be specifically informed of that policy prior to initiating treatment with PGT. Actual practice across individual clinics vary considerably but fall within these guidelines.

For example, a large IVF program in the United States recently performed a comparison of implantation rates between patients using PGT-A that elected to either transfer an embryo based on its sex or based on its morphological quality (24). The report indicated that 48% of patients in the study chose which embryo to transfer based upon its sex, illustrating that PGT-SS within PGT is a relatively common practice. PGT-P may be applied in a similar fashion. That is, patients may elect to select amongst available euploid embryos based upon additional information; the risk of polygenic disease.

ASRM has also released an ethics opinion on the use of PGT for adult onset conditions, indicating that reproductive liberty arguments ethically allow for testing conditions that are highly treatable, of lesser severity, or exhibit reduced penetrance (8). For example, alpha 1 antitrypsin, hereditary hemochromatosis, non-classic 21 hydroxylase deficiency, biotinidase deficiency, and familial Mediterranean fever all fit this category. These disorders are amongst the most frequently identified in the clinical setting through expanded carrier screening (25). In addition, mutations in the BRCA1/2 genes are a common indication for PGT-M, recently reported to represent ~4% of all PGT-M cases at a large reference laboratory (26). BRCA mutations account for ~5–6% of breast cancer (27, 28). However, polygenic origins may account for 10–15% given the frequency of familial breast cancer (29).

Remaining questions regarding the clinical utility of screening embryos for polygenic disease risk reduction include to what extent family history is required. Polygenic risk scoring in adults is known to benefit from availability of data on age, sex, and clinical risk (30, 31). Although the sex of an embryo is easily predicted, age, and clinical risk data are not available, which may necessitate family history as a surrogate (32).

While prior research focused on expected gain in polygenic trait value (height and intelligence) with embryo selection (7), the present study demonstrates the clinical utility of embryo selection for polygenic disease risk reduction. As new databases are developed, new predictors will continue to become available, and existing predictors may be further improved (Table 1). Prediction of risk for PCOS, endometriosis, and aneuploidy are of particular interest and relevance to patients with infertility, and represent an important focus of future research on polygenic risk scoring in embryos.

**Table 1:** Existing area under the curve (AUC) statistics for several complex diseases.

**Disease**	**AUC**	**References**
Type 1 diabetes	0.85–0.92	(14)
Type 2 diabetes	0.72^*^	(10)
Coronary artery disease	0.81^*^	(10)
Atrial fibrillation	0.77^*^	(10)
Heart attack	0.56–0.6	(33)
Hypercholesterolemia	0.628	(31)
Breast cancer	0.68^*^	(10)
Breast cancer	0.63	(34)
Malignant melanoma	0.58	(31)
Prostate cancer	0.646	(35)
Testicular cancer	0.65	(31)
Basal cell carcinoma	0.631	(31)

* *Includes age and sex*.

## Data Availability Statement

The datasets generated for this study will not be made publicly available due to maintaining confidentiality of identifiable patient data.

## Ethics Statement

Ethical review and approval was not required for the study on human participants in accordance with the local legislation and institutional requirements. The patients/participants provided their written informed consent to participate in this study. Written informed consent was obtained from the individual(s) for the publication of any potentially identifiable images or data included in this article.

## Author Contributions

NT, SH, EB, JH, and LT designed the study. JE and KP counseled the patient. NT, LT, EB, JE, LL, RZ, BR, and AS analyzed the data. RZ and BR performed the laboratory work. NT, JE, LL, and LT wrote the manuscript.

### Conflict of Interest

All authors are employees or founders of Genomic Prediction Inc.
