# Supplementary material for: Utility and First Clinical Application of Screening Embryos for Polygenic Disease Risk Reduction
Source: Front Endocrinol (Lausanne). 2019 Dec 4;10:845. doi: 10.3389/fendo.2019.00845 (PMC6915076; doi:10.3389/fendo.2019.00845)
Supplement: Supplementary file 1 [file Data_Sheet_1.docx]

**Supplementary Methods: Relative Risk Reduction (RRR)**

In this technical supplement, we present simulation results for assessment of relative risk reduction in cases of polygenic disease with embryo selection. The case considered is picking an embryo with the lowest polygenic risk score (PRS) in a batch of sibling embryos, comparing its incidence of disease to a randomly selected embryo.

**Introduction to RRR**

The liability-threshold model is widely used for binary disease traits in genetics (Lee et al., 2012; Lee et al., 2011; Wray and Visscher, 2015; Wray et al., 2010). In this model, disease status is completely determined by a continuous *liability* score exceeding a threshold *T* . In a case-control study, we say that the *i^th^* sample has liability

$$l_{i}=g_{i}+e_{i}$$

where *g_i_* is genetic liability score and *e_i_* an error term, and cases are those for which *l_i_ > T* . The genetic liability *g_i_* is chosen so that *g_i_* ∼ *N* (0*, h*^2^ )*,* where *h*^2^ is the heritability of liability and *g_i_* + *e_i_* ∼ *N* (0*,* 1). The threshold *T* is then chosen so that if *K* is the disease prevalence in the population and Φ is the cumulative distribution function of the normal distribution,

*L*

$$1-K=\Phi(T).$$

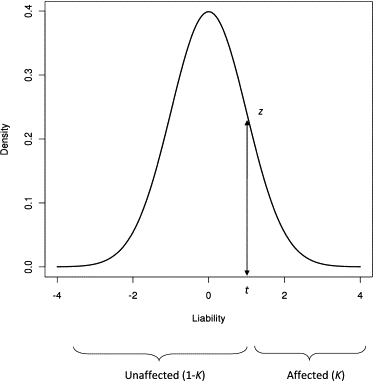


**Sibling Model**

To generate a realistic model for a group of siblings, we must correct for sibling relatedness. We must partition the portion of the liability scale captured by additive genetics, *A*, in two: genetic risk measured by the predictor, *g_p_* and unmeasured genetic risk *g_u_* which may also be correlated between relatives:

*A* = *g_p_* + *g_u_.*

Assuming the loci in the polygenic predictor are independent and have additive effects, the correlation between any pair of siblings’ measured genetic risk score *A* is 0.5. (To see this, by additivity it suffices to look at the correlation on a single allele from one parent. A simple calculation gives the desired result.) Suppose we have *k* siblings and mid-parental risk *µ* on the liability scale. Then the polygenic risk score vector for the siblings, *A*, is a multivariate normal with mean vector:

***µ*** ∼ (*µ, . . . , µ*)

And covariance:

$$\Sigma_{i,j}=\sigma_{g}^{2} ifi=j\frac{1}{2}\sigma_{g}^{2}ifi\neq j$$

We can generate $g_{u}$ in the same manner, with an unmeasured genetic risk score, having mean $r$ and covariance

$\Sigma_{i,j}=h^{2}-\sigma_{g}^{2} ifi=j\frac{1}{2}\left( h^{2}-\sigma_{g}^{2} \right)ifi\neq j$

where $h^{2}$ is the heritability of the disease on the liability scale.

**Family Model**

The relative risk for the lowest PRS embryo among $k$ sibling embryos necessarily depends on the parental risk score. As above, our model for liability is that a case is one where

$L_{i}=A_{i}+g_{u,i}+e_{i},$

exceeds threshold $T$, with $A$ the additive polygenic risk score, $g_{u}$ an unmeasured polygenic risk score for the $i$th sample, and $e_{i}$ an error term for the $i$th sample that is uncorrelated between samples:

$E\left[ e_{i}e_{j} \right]=0.$

One reasonable special case is a family where one parent has a polygenic risk score and unmeasured risk score equal to the mean of cases, i.e.

$A_{i}=E\left[ A|A+E>T \right]$

and

$g_{u,i}=E\left[ g_{u}|A+E>T \right].$

These parameters depend on $T$, $\sigma_{g}^{2}$ and $h^{2}$. To simplify the analysis, we will simply select $A_{1}=\sigma_{g}^{2}T$ and $g_{u,1}=\left( h^{2}-\sigma^{2} \right)T$. In this scenario, for a disease where there is little assortative mating, it is also reasonable to assume the other parent has mean risk: $A_{2}=0,g_{u,2}=0$. This means that the $k$ embryos will be drawn from the distribution:

$\mathbf{A}=N\left( \boldsymbol{\mu}_{\mathbf{A}},\boldsymbol{\Sigma}_{\mathbf{A}} \right)$

with $\boldsymbol{\mu}_{\mathbf{A}}=\left( \sigma_{g}^{2}\frac{T}{2},\cdots,\sigma_{g}^{2}\frac{T}{2} \right)$, and unmeasured genetic risk

$\mathbf{g}_{\mathbf{u}}=N\left( \boldsymbol{\mu}_{\mathbf{g}_{\mathbf{u}}},\boldsymbol{\Sigma}_{\mathbf{u}} \right)$

with

$\boldsymbol{\mu}_{\mathbf{g}_{\mathbf{u}}}=\left( \left( h^{2}-\sigma_{g}^{2} \right)\frac{T}{2},\cdots,\left( h^{2}-\sigma_{g}^{2} \right)\frac{T}{2} \right).$

From this point, we simply calculate the reduction in number of cases in the embryo with lowest polygenic risk score versus a randomly selected embryo.

**Estimating Parameters: From Observed Scale to Liability Scale**

There are two parameters to be estimated from observed data to: $h^{2}$ and $\sigma_{g}^{2}$. The former is the heritability of the trait on the liability scale, which is estimated from studies on the observed binary scale along with estimates of disease prevalence. Let $y$ denote phenotypic case-control status and $l$ the liability score. Then:

$cov(y,l\mathbb{)=E}\left[ y\cdot l \right]-\mathbb{E}\left[ y \right]\mathbb{E}\left[ l \right]=Ki$

where $K$ is the prevalence of the disease and $i$ is the mean liability for cases. On the other hand, Lee et. al [3] derive a formula for transforming the observed $R^{2}$ in a case-control study, $R_{cc}^{2}$, to the $R^{2}$ in a liability scale. Let $K$ be the prevalence of the disease in the population at large and $P$ is the ascertained prevalence in the case control study, and $T$ defined as the threshold above. We further define $z=\phi(T)$ , with $\phi$ the probability density of the normal distribution. Then the explained variance of the predictor in the liability scale is given by

$R_{l_{cc}}^{2}=\frac{R_{cc}^{2}C}{1+R_{cc}^{2}\theta}$

where

$C=\frac{K(1-K)}{z^{2}}\frac{K(1-K)}{P(1-P)},$

$\theta=\frac{z\left( P-K \right)}{K\left( 1-K \right)}\left( \frac{z\left( P-K \right)}{K\left( 1-K \right)}-T \right).$

For ease of notation we will denote this by $\sigma_{g}^{2}$.

**Results of Modeling Simulations**

Using type 1 diabetes as an example, we compute the threshold $T$ from the disease prevalence in a representative adult demographic (0.3%):

• $\Phi(1-.003)\approx2.74$

• $h^{2}=0.8$

We choose $k=7$ to match a typical number of embryos in a PGT-M case screening for a monogenic disorder. As validation of our model, we find that a randomly selected embryo is a case approximately 6% of the time, in line with sibling concordance studies of type 1 diabetes. In the simulation below, we allow the proportion of variance in the liability scale to vary.


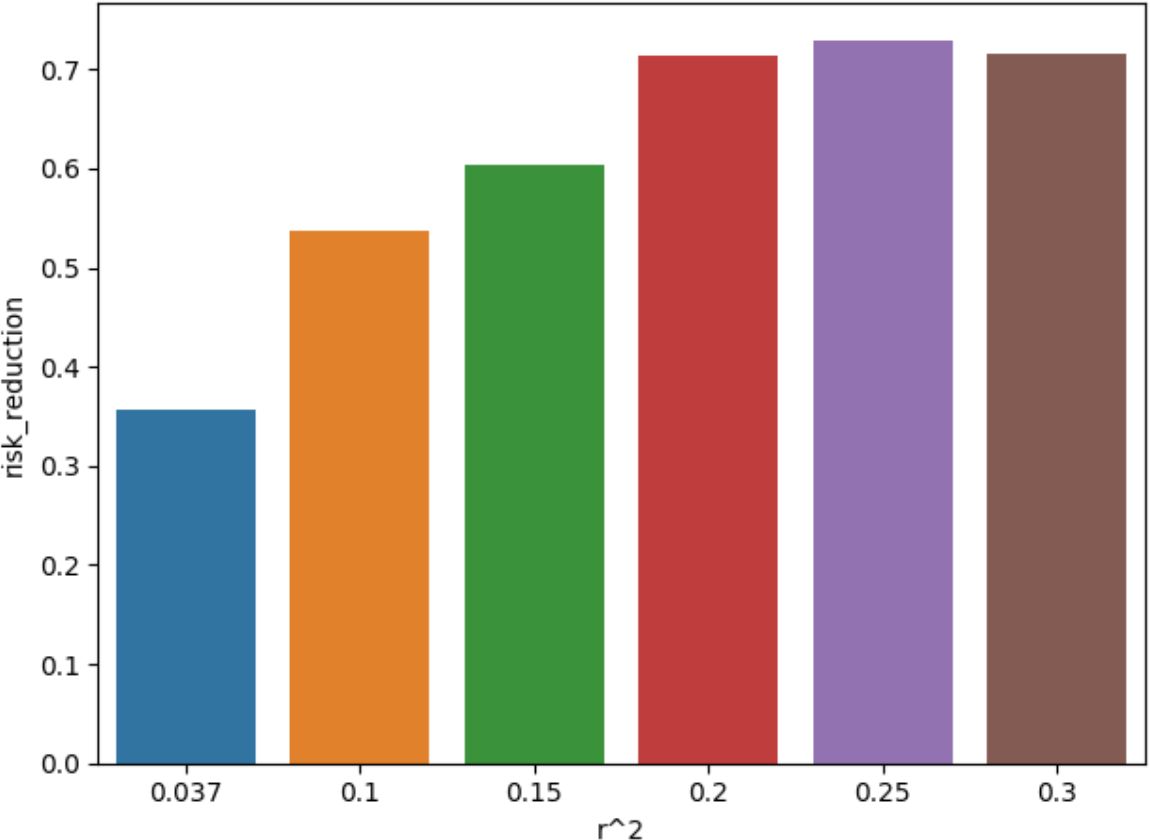

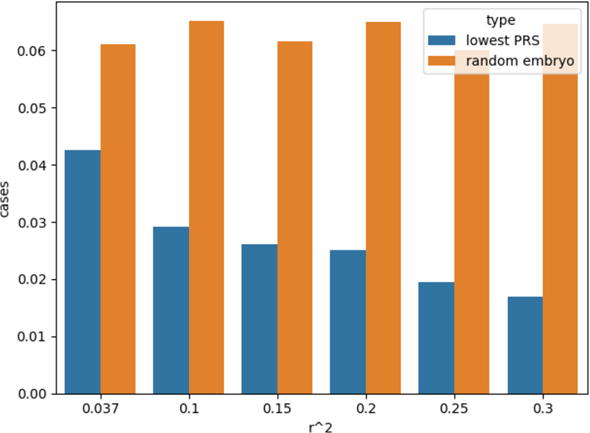


**Supplemental Figure 1**: Simulation output, describing relative reduced risk, with relative fraction of case embryos selected for implantation in situations of embryo screening vs chance, as a function of r^2.

**Supplemental Figure 2**: Simulation output, describing disease risk reduction to the child of the parent using screening to conceive, compared to selecting an embryo by chance, as a function of r^2.

**Supplementary References**

Lee, S.H., Goddard, M.E., Wray, N.R., Visscher, P.M., 2012. A better coefficient of determination for genetic profile analysis. Genet Epidemiol 36(3), 214-224.

Lee, S.H., Wray, N.R., Goddard, M.E., Visscher, P.M., 2011. Estimating missing heritability for disease from genome-wide association studies. Am J Hum Genet 88(3), 294-305.

Wray, N.R., Visscher, P.M., 2015. Quantitative genetics of disease traits. J Anim Breed Genet 132(2), 198-203.

Wray, N.R., Yang, J., Goddard, M.E., Visscher, P.M., 2010. The genetic interpretation of area under the ROC curve in genomic profiling. PLoS Genet 6(2), e1000864.
